# Plant Functional Traits, but Not Community Composition, Are Affected by Summer Precipitation and Herbivory in an Old‐Field Ecosystem

**DOI:** 10.1002/ece3.71399

**Published:** 2025-05-07

**Authors:** Julia N. Eckberg, Mariano A. Rodríguez‐Cabal, M. Noelia Barrios‐García, Nathan J. Sanders

**Affiliations:** ^1^ Department of Ecology and Evolutionary Biology University of Michigan Ann Arbor Michigan USA; ^2^ Grupo de Ecología de Invasiones, INIBIOMA Universidad Nacional del Comahue, CONICET San Carlos de Bariloche Río Negro Argentina; ^3^ Rubenstein School of Environment and Natural Resources University of Vermont Burlington Vermont USA; ^4^ CENAC‐APN, CONICET Universidad Nacional del Comahue (CRUB) San Carlos de Bariloche Argentina

**Keywords:** biomass, functional diversity, log response ratio, plant‐herbivore interactions

## Abstract

Both precipitation and herbivores can independently control plant community composition and ecosystem function. However, few studies have experimentally examined the potential interactive effects of altered precipitation and herbivores on plant communities and plant traits. Here, we manipulated summer precipitation and insect presence in an old‐field ecosystem and quantified their interactive effects on plant community structure and functional traits. Overall, the effect of an insect herbivore on the plant community was contingent on the precipitation treatment. There were no experimental effects on total plant biomass or plant species richness, but grass biomass was higher in the absence of insect herbivores only in reduced summer precipitation plots. Furthermore, plant functional diversity and the community‐averaged trends of several plant functional traits related to resource use and herbivore resistance varied systematically with reduced precipitation and insect presence. We demonstrate that the effect of reduced precipitation on plant biomass, functional diversity, and the community‐averaged trends of plant functional traits can be mediated by the presence of insects. Our findings further suggest that the functional traits of the common plant species in the community are the most affected by the combined manipulation of altered summer precipitation and insect presence.

## Graphical abstract

We demonstrate that the effect of reduced precipitation on plant biomass, functional diversity, and the community‐averaged trends of plant functional traits can be mediated by the presence of grasshoppers. Our findings further suggest that the functional traits of the most common species in the community are the most affected by the combined effects of reduced summer precipitation and the presence of a generalist herbivore.

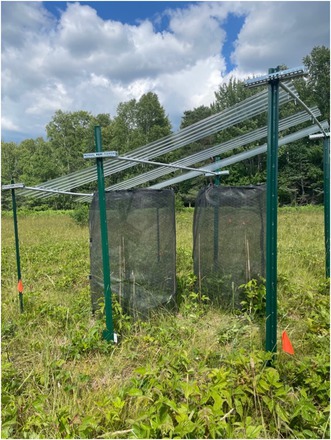

## 1 Introduction

Climate strongly influences the composition and function of plant communities (Cleland et al. 2013; Gherardi and Sala 2019; Liu et al. 2020; Zhou, Wang, et al. 2023; Zhu et al. 2024). Critically, climate change is causing precipitation regimes to shift in regions around the world (Wuebbles et al. 2019; Zscheischler et al. 2020; Zhou, Yu, et al. 2023). Shifts in precipitation alter water availability, which directly affects plant growth and species persistence (Knapp et al. 2001; Alon and Sternberg 2019; Smith et al. 2020; Luong et al. 2021), with the potential to drive community‐level shifts in composition and modify ecosystem function (Zeppel et al. 2014; Li et al. 2020; Zhang and Xi 2021; Ónodi et al. 2025). Yet, the response of plant communities to changes in precipitation is often idiosyncratic and can depend on a variety of community and ecosystem characteristics (Knapp et al. 2008, 2015). For example, several studies have demonstrated that reduced precipitation generally leads to a decline in plant biomass (Kardol et al. 2010; Cantarel et al. 2013; Zang et al. 2020), while others have reported no change in plant biomass (Frank 2007; Cherwin and Knapp 2012). Plant species richness can also decrease (Tilman and El Haddi 1992; Copeland et al. 2016), increase (Smith et al. 2020), or remain the same (Hoover et al. 2014) in response to reduced precipitation. The variability in plant community response to altered precipitation underscores the importance of uncovering the role of local interactions in mediating the plant community response to climate change.

While the effects of altered precipitation on plant communities are often determined by quantifying differences in plant biomass or taxonomic diversity (Hoover et al. 2014; Batbaatar et al. 2022), altered precipitation can also drive changes in plant functional traits (Griffin‐Nolan et al. 2019). Changes in functional traits in response to precipitation, or any environmental driver for that matter, arise from several potential mechanisms. For example, reduced precipitation can act as an agent of selection, filtering out those species which lack the functional traits that allow them to persist under drier conditions (Caruso et al. 2020). Alternatively, some species might exhibit phenotypic plasticity and differentially express a trait under the altered precipitation regime (Pérez‐Ramos et al. 2019). For instance, increased precipitation often correlates with increased specific leaf area (SLA), plant height, and leaf dry matter content (LDMC) (Moles et al. 2009; Sandel et al. 2010; Cheng et al. 2021). Specific leaf area characterizes plant carbon gain relative to water loss (Liu et al. 2017) and is closely tied to precipitation and water availability because while SLA positively correlates with plant growth rate (Galmes et al. 2005), reduced SLA when water is limited can improve water use efficiency (Liu and Stützel 2004). Plant height, like SLA, positively correlates with growth rate (Pérez‐Harguindeguy et al. 2013), and as such when precipitation causes water to be abundant, plants can maximize growth rate and increase height (Moles et al. 2009). Leaf dry matter content characterizes leaf tissue density (Pérez‐Harguindeguy et al. 2013). High LDMC provides resistance to herbivory as well as structural drought tolerance and can positively correlate to physiological drought tolerance (Westbrook et al. 2011; Blumenthal et al. 2020), making investment in high LDMC an important strategy to conserve water when precipitation is reduced and water is limited.

The effect of reduced precipitation on plant communities and traits may be mediated by other factors, such as herbivory by insects. Like altered precipitation regimes, insect herbivores can also have a strong influence on plant community structure and ecosystem function (Crawley 1989; Carson and Root 1999; Smith et al. 2020; Agrawal and Maron 2022). The effects of insect herbivores on plant communities and ecosystem function, like precipitation, tend to vary among studies. While some studies report that insects have a positive effect on plant biomass (Belovsky and Slade 2000; Stein et al. 2010; Garcia and Eubanks 2019), other studies report a negative (Carson and Root 1999; Stein et al. 2010; Smith et al. 2020), or no effect (Stein et al. 2010; Blue et al. 2011) of insects on plant biomass. One mechanism by which insects can directly affect plant biomass is by consuming plant tissue (Agrawal and Maron 2022), as insect herbivores can remove up to 15% of plant biomass in terrestrial plant communities and ultimately reduce aboveground plant biomass (Kozlov and Zvereva 2018). Insects can also modify plant biomass indirectly by altering nutrient cycling. For example, insects can promote nitrogen cycling and ultimately facilitate increased plant biomass by modifying the quantity and quality of plant litter (Belovsky and Slade 2000). Similarly to plant biomass, the effect of insects on plant species richness is also inconsistent among studies, with reports of positive (Korell et al. 2019), negative (Hendrix et al. 1988; Stein et al. 2010; Smith et al. 2020; Agrawal and Maron 2022), and no effect (Hendrix et al. 1988; Kim et al. 2015) of reduced insect abundance on plant richness. One mechanism by which insects can affect plant species richness is by altering interactions among plant species through selective feeding. For example, if insects selectively consume the dominant plant species and reduce the competitive effect of that dominant species, insects can promote species coexistence (Carson and Root 2000; Agrawal and Maron 2022). Additionally, coexistence theory posits that insect herbivores can reduce fitness differences between plant species and facilitate species persistence, which can ultimately result in greater plant diversity (HilleRisLambers et al. 2012; Schmidt et al. 2020). The discordant effect of insect herbivores on plant communities might ultimately result from other factors influencing insect communities. For example, precipitation can affect herbivore abundance and diversity (Jamieson et al. 2012; Zhu et al. 2014), which may determine the magnitude and direction of the effect of insects on plant communities.

Along with plant biomass and diversity, insects can also shape plant traits and functional composition by acting as an agent of selection or inducing a plastic response in some species. For example, insects can directly alter plant traits by selectively consuming plants with either thin, less tough leaves that are easier to eat (Schädler et al. 2003) or plants with leaves of a higher nutrient concentration (Schmitz 2008a, 2008b). For example, grasses tend to have high nitrogen concentrations (Schmitz 2008a, 2008b; Rosenblatt 2021) and the preferential consumption of grasses by insects can ultimately affect the relative biomass of different plant functional groups in a community (Schmitz 2003). While the primary mechanism by which insects affect plant traits is selective feeding, insects can also shape plant traits and functional composition indirectly by altering soil properties (Metcalfe et al. 2014).

Notably, most studies on the effects of precipitation and insect herbivory on plant communities focus on these factors in isolation; indeed, there are several reviews on each of their effects (Crawley 1989; Weltzin et al. 2003; Knapp et al. 2008; Reyer et al. 2013; Zeppel et al. 2014; Kozlov and Zvereva 2018; Agrawal and Maron 2022). Though precipitation and insect herbivores independently structure plant communities, their potentially interactive effects on communities and ecosystems are less well understood or studied. Given the variable effects of precipitation on plant diversity and biomass across studies (i.e., Kardol et al. 2010; Cherwin and Knapp 2012; Cantarel et al. 2013; Zang et al. 2020; Smith et al. 2020), it suggests that the effects of precipitation on plant communities are often context dependent, and it could be that interactions with insects mediate the effect of altered precipitation on plant communities. Importantly, recent evidence suggests that insects do mediate the effect of altered precipitation on plant communities (Xu et al. 2021; Luo et al. 2024). For example, insects can facilitate a higher abundance of drought‐resistant plant species and subsequently maintain total plant abundance during drought (Xu et al. 2021). To further explore the role of insects in shaping plant community response to altered precipitation, in this study we test the interactive effects of reduced summer precipitation and the presence of a single generalist herbivore on plant biomass, species richness, functional diversity, and the community‐averaged trends of a suite of plant functional traits by factorially manipulating summer precipitation and herbivore presence in an old‐field ecosystem. Specifically, we ask the following interrelated questions:

1. Are there interactive effects of reduced summer precipitation and herbivore presence on plant species richness or aboveground plant biomass? Do the effects of reduced summer precipitation and herbivore presence vary among grasses, forbs, and shrubs?
2. Are there interactive effects of reduced summer precipitation and herbivore presence on plant functional diversity and the community weighted means of a suite of plant functional traits?

## 2 Materials and Methods

### 2.1 Study System

We conducted this experiment in an old field at the University of Michigan Biological Station near Pellston, Michigan (45.558° N, −84.650° W). We chose to conduct our experiment in an old field because old‐field plant and insect communities are relatively easy to manipulate, as evidenced by the numerous manipulative experiments studying the effects of plant‐insect interactions and climate on old‐field ecosystems (Carson and Root 1999, 2000; Barton et al. 2009; Engel et al. 2009; Rosenblatt 2021). Mean annual precipitation at the site is 766 mm and mean annual temperature is 6.7°C (Climate Data, 2024). Agricultural practices ended at this site in 1938, and it was clear cut in 1991 to return it to an early successional state. The site is mowed semiannually to maintain an old‐field ecosystem. The plant community primarily consists of perennial, herbaceous species. The four most abundant species are *Rubus flagellaris* (shrub), *Poa pratensis* (grass), *Pilosella caespitosa* (forb), and *Symphyotrichum urophyllum* (forb). *Melanoplus femurrubrum* (Red‐legged grasshopper) is a generalist, leaf‐chewing insect herbivore commonly found at our study site and in surrounding old fields. We selected *M. femurrubrum* as our study species because there is a large body of work investigating the effect of *M. femurrubrum* on plant communities (Schmitz 1998, 2008a, 2008b; Rosenblatt 2021), and *M. femurrubrum* are distributed across North America and are commonly found in old fields (Capinera 2020). Despite having a wide dietary breadth, *Melanoplus* grasshoppers preferentially consume grasses, which have higher nitrogen concentrations relative to other plant groups, to meet metabolic demands (Capinera 2020; Rosenblatt 2021). Importantly, grasshoppers are generally the dominant insect herbivore in most old‐field ecosystems (Branson et al. 2006), and their effects on plant community structure and ecosystem function can be comparable to that of the whole insect community (Schmitz 2008a). Therefore, by studying an experimentally tractable dominant herbivore like *M. femurrubrum* , it is possible to gain an understanding of the role of insect herbivores more broadly in structuring plant communities and ecosystem processes.

### 2.2 Precipitation Treatment

To test the effect of reduced summer precipitation on the plant community, we established 16 2 × 2 m experimental plots in May 2023. Our experimental plots were 2 × 2 m because our plots needed to be large enough to contain multiple subplots, and this is a similar size to other experiments manipulating incoming precipitation (Yahdjian and Sala 2002; Gherardi and Sala 2013, 2019). For each 2 × 2 m plot, we assigned one of two precipitation treatments: (1) reduced summer precipitation and (2) ambient precipitation. We are particularly interested in reducing summer precipitation because, in northern Michigan, summer precipitation is projected to decline by up to 36% in the coming decades (Kunkel et al. 2022). In our experiment, we designed rainout shelters to intercept 50% of incoming summer precipitation starting in June 2023 using a design modified from Rudgers et al. (2023). We chose to intercept 50% of summer precipitation in order to assess the response of plant communities to a more severe, but still plausible, reduction in summer precipitation. Rainout shelters consisted of four metal U‐posts driven into the ground, with two posts ~2.13‐m tall and two posts ~1.52‐m tall to create a sloped roof. We constructed the roof using three sheets of corrugated polycarbonate plastic sheets that allow for 93% light transmission (Tuftex; 33.02‐cm wide, 243.84‐cm long). We attached roof panels to PVC conduits using nuts and bolts, with both aluminum and rubber washers on both ends to stabilize and protect panels from damage. We cut holes in roof panels for plots assigned the ambient precipitation treatment to account for potential rainout shelter effects while allowing for precipitation to reach the soil. We manipulated summer precipitation from the end of June until mid‐October to ensure we reduced precipitation through the end of the growing season. We collected data in 2023 from August 8 to 14, which corresponds to the peak of the growing season. Since we collected plant biomass, richness, and functional trait data during the peak growing season, we manipulated summer precipitation for 47 days prior to data collection, and for 25 of those days, soil volumetric water content (%; VWC) was statistically higher in ambient precipitation plots relative to reduced precipitation plots by, on average, 4% (±SD 1.99; Appendix S1: Table S1).

### 2.3 Grasshopper Treatment

**FIGURE 1:**
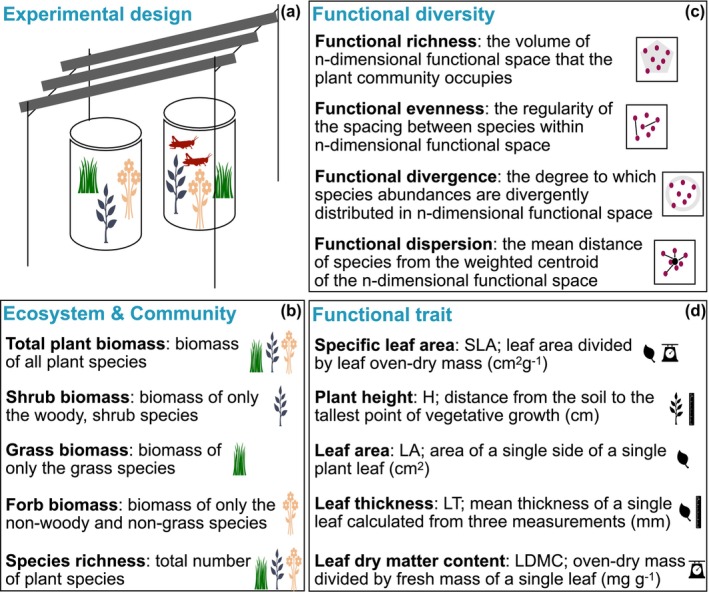
A schematic representation of the rainout shelter and insect enclosure plot setup (a), along with a description of each ecosystem and community (b), functional diversity (c), and functional trait (d) measure included in the study. The graphs that accompany the functional diversity definitions are adapted from Legras et al. (2018). Functional diversity definitions are adapted from Mason et al. (2005) and Luo et al. (2023). Functional trait definitions are adapted from Pérez‐Harguindeguy et al. (2013).

To test the effect of grasshopper presence on the plant community, underneath each 2 × 2 m rainout shelter we constructed two paired, cylindrical 1‐m^2^ enclosures in May 2023 following Schmitz et al. (2004). We constructed enclosures using Gray Steel Chicken Wire (Garden Craft; 2.54 cm mesh) and then covered enclosures with Crystal Clear Charcoal Fiberglass Screen Mesh (M‐D) (Appendix S1: Figure S1). Each enclosure was 1.2 m tall, and we buried the base of each enclosure at least 10 cm into the soil to ensure enclosures were fully sealed and insects could not move into or out of enclosures. After sealing the enclosures, we used a leaf blower modified to suck up insects to remove insects from all enclosures in mid‐May. We then manipulated grasshopper presence at two levels within each pair of 1‐m^2^ enclosures: (1) all insects removed from mid‐May to the end of the growing season, and (2) the introduction of six *Melanoplus femurrubrum* (Red‐legged grasshopper) in Mid‐July. In mid‐July, we collected *M. femurrubrum* grasshoppers at the study site and from a nearby old field using a sweep net and added six individuals to each enclosure assigned the grasshoppers present treatment. The standard density of *M. femurrubrum* in this old field is three individuals per 1‐m^2^ (unpublished data), so we stocked enclosures with six *M. femurrubrum* to account for any potential deaths caused by stress during the translocation process. By constructing enclosures underneath each rainout shelter, we employed a split‐plot design that reduced summer precipitation and modified grasshopper presence (Figure 1).

### 2.4 Data Collection

To assess the potential interactive effects of reduced summer precipitation and grasshopper presence on the plant community, we first identified all plant species in each enclosure in mid‐August and visually estimated the percent cover of each species in each enclosure. We then quantified treatment effects on aboveground plant biomass by clipping all plant species in a 0.5 × 0.5 m section of each enclosure at the soil level. After clipping plants, we sorted plants by plant functional group (grass, shrub, or forb) and oven‐dried them at 60°C for at least 48 h and then weighed them. We also measured plant functional traits in 24 of the 32 enclosures, encompassing eight replicates of each experimental treatment combination (reduced precipitation, grasshoppers present; reduced precipitation, grasshoppers absent; ambient precipitation, grasshoppers present; ambient precipitation, grasshoppers absent). Due to time constraints, we were not able to measure traits in all of the enclosures. For each enclosure included in trait analysis, the total percent cover of all species selected for trait measurements was at least 80% and no more than five species were included in trait analysis. We measured five plant functional traits: plant height (H), leaf area (LA), leaf thickness (LT), SLA, and LDMC (Appendix S1: Figure S2). We selected these traits because they relate to resource use (H, LA, SLA) and herbivore resistance (LT, LDMC), and were therefore likely to be directly affected by our experimental treatments (Pérez‐Harguindeguy et al. 2013; Kramp et al. 2022). For each species included in the functional trait analysis, we measured traits for three randomly selected individuals in each enclosure and calculated the average trait value to provide trait data at the species level. For each individual, we measured plant height (cm) to the nearest 0.5 cm using a meter stick in the field (Pérez‐Harguindeguy et al. 2013). We then collected one leaf from each individual, wrapped the petiole in a wet paper towel, and stored it in a refrigerator to keep leaf tissue fresh for further measurements. Within 24 h of collection, we scanned leaves (Epson Perfection V19) and used the scanned leaf images to calculate LA (cm^2^) using imageJ (Schneider et al. 2012; Pérez‐Harguindeguy et al. 2013). Immediately after scanning leaves, we used digital calipers to measure LT (mm) at three different points on each leaf, avoiding the midrib, and calculated mean LT (referred to as LT hereafter) using the three measurements (Pérez‐Harguindeguy et al. 2013). We then weighed each leaf to the nearest 0.01 g, oven‐dried the leaves at 60°C for at least 48 h, and then reweighed them. We calculated SLA (cm^2^ g^−1^) as LA divided by oven‐dry leaf mass and LDMC (g g^−1^) as oven‐dry leaf mass divided by fresh leaf mass (Pérez‐Harguindeguy et al. 2013).

We measured soil volumetric water content (VWC; %) at a depth of 6 cm in each 2 × 2 plot throughout the growing season using TMS‐4 dataloggers (Wild et al. 2019). We deployed one TMS‐4 datalogger underneath each rainout shelter outside of the insect enclosures in order to quantify shelter effects on soil moisture independent of insect presence. Each TMS‐4 datalogger measured soil VWC every 15 min from deployment in the field in mid‐May to the end of August 2023.

### 2.5 Data Analysis

Before testing for experimental treatment effects on plant functional diversity and functional traits, we characterized the functional space of the plant communities by calculating the following functional diversity metrics for each insect enclosure: functional richness (the volume of n‐dimensional functional space that the plant community occupies), functional evenness (the regularity of the spacing between species within n‐dimensional functional space), functional divergence (the degree to which species abundances are divergently distributed in n‐dimensional functional space), functional dispersion (the mean distance of species from the weighted centroid of the n‐dimensional functional space; Mason et al. 2005; Laliberté and Legendre 2010; Luo et al. 2023). We also calculated the community weighted mean (CWM) of each trait by weighting the measured trait value by the plant species percent cover for all traits and plant species in each enclosure (Lepš and de Bello 2023). We used species percent cover as a proxy for abundance, a common practice when calculating functional diversity and trait CWM (Lepš et al. 2006; Lavorel et al. 2008). We performed all functional diversity and CWM calculations using the *dbFD* function in the *“FD”* R package (Laliberté and Legendre 2010) in R (R Core Team 2022).

To quantify the interactive effects of reduced summer precipitation and grasshopper presence on the plant community, we calculated a log response ratio for each plant response variable we measured (total plant biomass, shrub biomass, grass biomass, forb biomass, species richness, functional richness, functional evenness, functional divergence, functional dispersion, CWM H, CWM LA, CWM LT, CWM SLA, CWM LDMC) between paired grasshoppers present and grasshoppers absent enclosures within each 2 × 2 m plot. We used the following equation to calculate log response ratios:

$$\text{lnRR}=\ln\left(\frac{\text{Grasshoppers present}}{\text{Grasshoppers absent}}\right)$$

We then calculated the mean log response ratio and 95% confidence intervals of each variable for reduced summer precipitation and ambient precipitation plots using the *qnorm* function in the *“stats”* R package (R Core Team 2022). We identified the interactive effect of reduced precipitation and grasshopper presence on a plant community variable based on whether the 95% confidence interval for either ambient or reduced precipitation plots crossed the zero line. Log response ratios allow us to account for the paired nature of our experimental design wherein grasshoppers present and grasshoppers absent enclosures are paired underneath each 2 × 2‐m rainout shelter. Furthermore, effect sizes calculated as log response ratios are commonly employed to detect the independent and interactive effects of experimental manipulations on communities and ecosystems (Midolo et al. 2019; Gao and Carmel 2020; Zhang and Xi 2021; Shi et al. 2022; Toledo et al. 2023). We conducted all statistical analyses using R version 4.1.3 (R Core Team 2022).

## 3 Results

**FIGURE 2:**
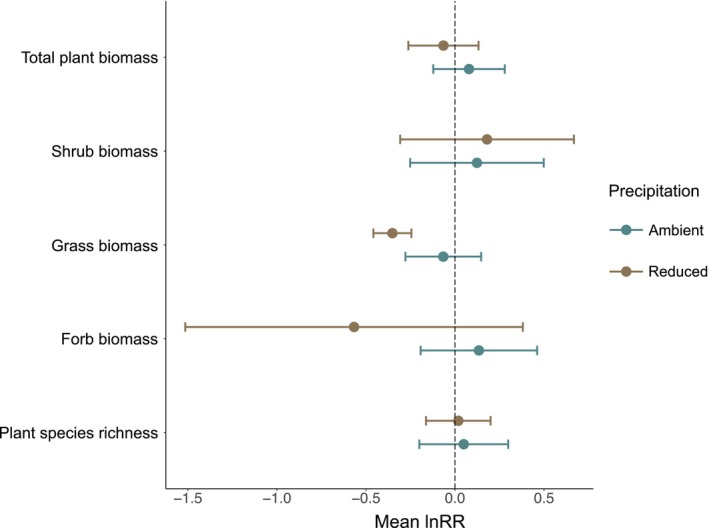
The effect of reduced summer precipitation and grasshopper presence on total plant biomass (g m^−2^), shrub biomass (g m^−2^), grass biomass (g m^−2^), forb biomass (g m^−2^), and plant species richness. Negative mean log response ratio (lnRR) indicates higher values of response variables in grasshoppers absent enclosures relative to grasshoppers present enclosures. Points indicate mean lnRR and lines indicate the 95% confidence interval. Blue points and lines represent mean lnRR and 95% confidence interval for ambient precipitation plots, brown points and lines represent mean lnRR and 95% confidence interval for reduced summer precipitation plots.

The biomass of grasses varied with reduced summer precipitation and grasshopper presence. Grass biomass was 18% higher in the absence of grasshoppers (45.15 g m^−2^ ± 25.71) compared to treatments with grasshoppers present (38.33 g m^−2^ ± 13.83), but this effect was observed only in the reduced precipitation plots (Figure 2). There were no interactive effects of reduced summer precipitation and grasshopper presence on total plant biomass, shrub biomass, forb biomass, or plant species richness.

**FIGURE 3:**
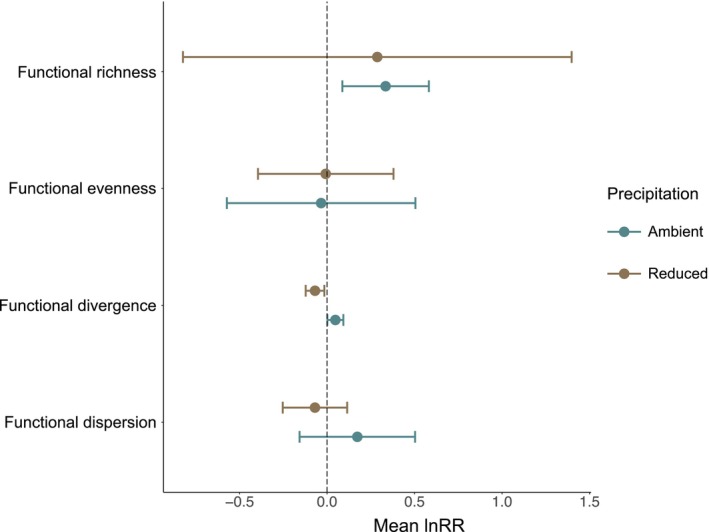
The effect of reduced summer precipitation and grasshopper presence on plant functional richness, functional evenness, functional divergence, and functional dispersion. Negative mean log response ratio (lnRR) indicates higher values of response variables in grasshoppers absent enclosures relative to grasshoppers present enclosures. Points indicate mean lnRR and lines indicate the 95% confidence interval. Blue points and lines represent mean lnRR and 95% confidence interval for ambient precipitation plots, brown points and lines represent mean lnRR and 95% confidence interval for reduced summer precipitation plots.

Functional richness and functional divergence varied with reduced summer precipitation and grasshopper presence, but there were no interactive effects on functional evenness or functional dispersion. Functional richness was 37% higher in the presence of grasshoppers (5.99 ± 0.83) compared to when grasshoppers were absent (4.38 ± 1.72), but this effect was observed only in ambient precipitation plots (Figure 3). Functional divergence was 7% higher in the absence of grasshoppers (0.88 ± 0.04) compared to when grasshoppers were present (0.83 ± 0.06), but this effect was observed only in reduced precipitation plots (Figure 3).

**FIGURE 4:**
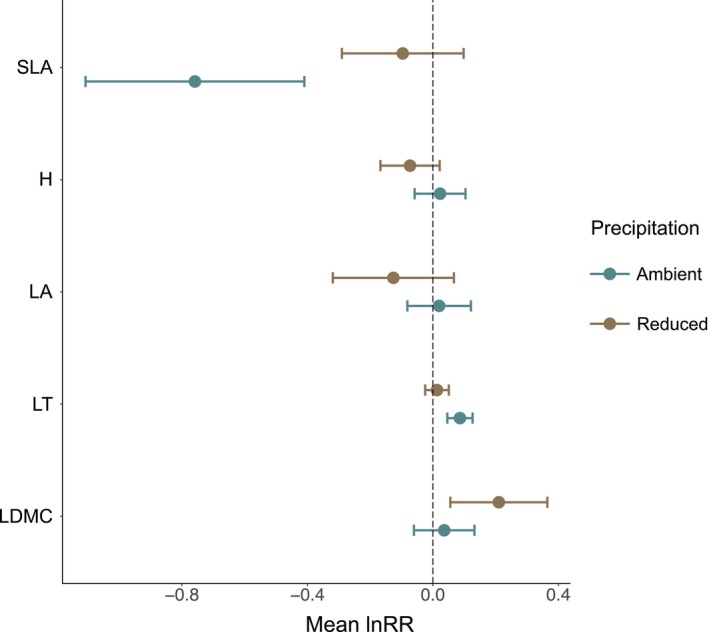
The effect of reduced summer precipitation and grasshopper presence on the CWM of specific leaf area (SLA; cm^2^ g^−1^), plant height (H; cm), leaf area (LA; cm^2^), mean leaf thickness (LT; mm), and leaf dry matter content (LDMC; g g^−1^). Negative mean log response ratio (lnRR) indicates higher values of response variables in grasshoppers absent enclosures relative to grasshoppers present enclosures. Points indicate mean lnRR and lines indicate the 95% confidence interval. Blue points and lines represent mean lnRR and 95% confidence interval for ambient precipitation plots, brown points and lines represent mean lnRR and 95% confidence interval for reduced summer precipitation plots.

The CWM of SLA, LT, and LDMC varied with reduced summer precipitation and grasshopper presence, but there were no effects on H or LA. Specifically, SLA was 147% higher in the absence of grasshoppers (242.12 cm^2^ g^−1^ ± 70.2) compared to when grasshoppers were present (98.04 cm^2^ g^−1^ ± 30.35), but this effect was observed only in ambient precipitation plots (Figure 4). Similarly, LT was 8% higher in the presence of grasshoppers (0.21 mm ± 0.006) compared to when grasshoppers were absent (0.19 mm ± 0.01), but only in ambient precipitation plots (Figure 4). LDMC was 25% higher when grasshoppers were present (0.75 g g^−1^ ± 0.1) compared to when grasshoppers were absent (0.60 g g^−1^ ± 0.06), but only in reduced precipitation plots (Figure 4).

## 4 Discussion

In this study, we documented the interactive effect of reduced summer precipitation and grasshopper presence on the community averaged trends of several plant functional traits, with fewer interactive effects on plant biomass, species richness, and functional diversity. Plant community functional traits, unlike plant biomass, species richness, and functional diversity, are highly sensitive to the abundance and traits of the most common plant species (Lavorel et al. 2008). Given that generalist insect herbivores typically consume the most common plant species in a community (Carson and Root 1999; Stein et al. 2010), our work suggests that grasshoppers mediate the effect of reduced precipitation on the plant community by modifying the abundance and traits of the most common species in the community. Furthermore, the Mass Ratio Hypothesis (Grime 1998) posits that the most common plant species alter community structure and drive the rate of ecosystem processes as a function of their large biomass. In line with the Mass Ratio Hypothesis, we highlight the importance of considering changes in the abundance or traits of the most common plant species in a community when predicting community‐level effects of environmental changes. Moreover, by demonstrating that the presence of a generalist insect herbivore can modulate plant community response to altered precipitation, our study underscores the need to incorporate biotic interactions across trophic levels into research that aims to predict plant community responses to climate change.

In addition to plant traits, reduced summer precipitation and grasshoppers interactively affected the biomass of grasses. Specifically, grass biomass was higher where grasshoppers were absent only when summer precipitation was reduced. In contrast, Schmitz (2006) found that *Poa pratensis* (Kentucky bluegrass) biomass was higher when grasshoppers were absent under ambient precipitation conditions. Our results may vary because of differences in plant community composition between our study sites. For example, the most abundant species at the Schmitz (2006) study site was an herbaceous forb ( *Solidago rugosa* ) while the most abundant species at our study site was a shrub *(Rubus flagellaris* ), and differences in the relative abundance of plant species between plant communities can alter community response to altered precipitation (Knapp et al. 2015). We should also consider that our reduced summer precipitation treatment still allowed for precipitation inputs, and we suggest that grasses may have rapidly taken up this water before it could infiltrate deeper soil layers, where forbs or shrubs more readily uptake water. As a result, our precipitation reduction likely favored shallow‐rooted grass species that are more primed to capture water from these smaller precipitation inputs that occupy the soil surface and evaporate quickly (Schwinning and Sala 2004; Fry et al. 2018). However, because grasshoppers preferentially consume grasses over forbs or shrubs, their presence may have offset this advantage (Schmitz 2008a, 2008b; Rosenblatt 2021). The lack of interactive effect of reduced summer precipitation and grasshopper presence on total plant biomass suggests that the interactive effects on grass biomass were not sufficient enough to manifest at the total biomass level.

Similarly to total plant biomass, there were no interactive effects of reduced summer precipitation and grasshopper presence on plant species richness. Though precipitation and insect herbivory can independently shape plant species richness (Carson and Root 1999; Harrison et al. 2020; Smith et al. 2020; Korell et al. 2021), another experiment in an old field found that grasshopper presence reduced aboveground biomass but did not affect species richness (Schmitz 2003).

Here, reduced summer precipitation and grasshopper herbivory interactively shaped plant functional richness and functional divergence. Specifically, plant functional richness was higher when grasshoppers were present only in ambient precipitation plots. Zuo et al. (2021) observed that in grass‐dominated ecosystems, plant functional richness tends to decline with increased precipitation. The negative relationship between precipitation and functional richness could relate to the stress gradient hypothesis wherein competitive interactions are more prevalent in low‐stress environments, which can lead to the loss of rare species and traits from communities (Bertness and Callaway 1994). We highlight the role of a generalist herbivore in mediating the relationship between precipitation and plant functional richness. We suggest that the presence of grasshoppers in ambient precipitation, low environmental stress conditions can reduce the strength of competitive interactions among plant species and subsequently facilitate the persistence of rare plant species, thereby increasing plant functional richness (Maestre et al. 2009). We observed the opposite trend in functional divergence where plant functional divergence was higher in reduced summer precipitation plots only when grasshoppers were absent. Another experiment observed a positive correlation between precipitation and the divergence of plant traits (Zuo et al. 2021), suggesting that reduced precipitation may operate as an abiotic filter and constrain the diversity of functions within the plant community (MacArthur and Levins 1967). Notably, reduced precipitation and grasshopper herbivory both stress the plant community (Suzuki et al. 2014), and as such, both precipitation and herbivory have the potential to filter and limit plant functional divergence (Bernard‐Verdier et al. 2012; Jäschke et al. 2020). In our experiment, when we reduced precipitation but removed grasshoppers, we effectively eliminated one stressor that could constrain functional divergence. As a result, the plant community had a relatively higher diversity of functions when we reduced precipitation but removed grasshoppers compared to when the plant community was experiencing both reduced precipitation and grasshopper herbivory. Overall, grasshoppers increased functional richness under ambient precipitation conditions but reduced functional divergence when summer precipitation was reduced. We suggest that under ambient precipitation, grasshoppers promote functional richness by reducing the strength of competitive interactions, and when precipitation is reduced, the combined stress of altered precipitation and herbivory strengthens abiotic and biotic filtering, thus reducing functional divergence.

While we observed interactive effects of reduced summer precipitation and grasshopper presence on functional richness and functional divergence, there were no interactive effects on functional dispersion or functional evenness. Over longer time scales, functional dispersion in herbaceous communities can decline with reduced precipitation (Harrison et al. 2020), which may relate to the stress tolerance hypothesis wherein a narrower range of plant functional strategies are successful in harsher climates (Currie et al. 2004). Our experiment was conducted over the course of a single growing season, while several other studies that observed a correlation between precipitation and functional dispersion occurred for longer time periods (Harrison et al. 2020; Zuo et al. 2021; Wang et al. 2022). Other studies similarly did not observe any effects on plant functional diversity over short time scales (Spasojevic et al. 2014; Harrison et al. 2020).

Altered summer precipitation and grasshopper herbivory interactively affected the CWM of several functional traits related to resource use and herbivore resistance. In ambient precipitation plots, SLA was higher only when grasshoppers were absent. Notably, plant community composition did not vary with reduced summer precipitation or grasshopper presence (Appendix S1: Figure S3), which suggests overall that differences in the CWM of plant traits between treatments were driven by intraspecific trait variation rather than species turnover. High SLA is a hallmark trait of resource acquisitive plant species (Wright et al. 2004; Pérez‐Harguindeguy et al. 2013), and resource acquisitive species tend to be more susceptible to insect herbivory in part because they typically invest fewer resources in physical or chemical defenses (Züst and Agrawal 2017; Descombes et al. 2020). Though SLA can correlate with increased herbivory (Pérez‐Harguindeguy et al. 2013), SLA also tends to increase with increased precipitation (Sandel et al. 2010; Dwyer et al. 2014), which is in line with our findings of higher SLA under ambient precipitation conditions. Furthermore, high SLA enables plants to maximize resource capture and growth when resources are abundant (Kooyers 2015; Griffin‐Nolan, 2019), a strategy that may be particularly effective when resource limitation occurs later in the growing season, as in our study. The interactive effect of reduced summer precipitation and grasshopper presence on SLA could indicate that plants with high SLA are better able to maximize water capture when precipitation is reduced, but only when the top‐down effects of grasshoppers are removed. Furthermore, resource acquisitive species with high SLA tend to be better competitors for shared resources (Wright et al. 2004; Pérez‐Harguindeguy et al. 2013), making SLA a particularly relevant trait to consider when trying to understand the role of traits in shaping species interactions and community composition. Though we observed no difference in species richness among our experimental manipulations, having high SLA when resources are abundant and herbivory is limited could, over time, ultimately lead to shifts in species abundance or richness through differences in competitive ability.

Leaf dry matter content can also correlate with tolerance to low soil moisture (Pérez‐Harguindeguy et al. 2013; Bongers et al. 2017; Blumenthal et al. 2020) and resistance to insect herbivory (Reese et al. 2016; Descombes et al. 2017; Blumenthal et al. 2020). LDMC can positively correlate with precipitation (Sandel et al. 2010; Xiyuan et al. 2018) or have no correlation with precipitation (Cheng et al. 2021). Here, given that reduced summer precipitation resulted in higher LDMC only when grasshoppers were present, we suggest that insect herbivores mediate the effect of reduced precipitation on LDMC.

Leaf thickness, like LDMC, is also associated with resistance to insect herbivores (Caldwell et al. 2016), but in contrast to LDMC, LT was higher in the presence of grasshoppers only in ambient precipitation plots. Thicker plant leaves tend to be tougher and more difficult for herbivores to consume (Westbrook et al. 2011), so our results suggest that grasshoppers selectively consume plants with thin, less tough leaves, which ultimately led to higher LT where grasshoppers were present. We may observe this trend only in ambient precipitation plots because when plants become dehydrated, their leaves shrink and LT declines (Scoffoni et al. 2014). Indeed, previous work has shown that LT often positively correlates with precipitation, such that when precipitation decreases, so does LT (Niinemets 2001; Phoenix et al. 2001; Song et al. 2024). If reduced precipitation selects for reduced LT while grasshoppers select for increased LT, then their effects may cancel each other out and ultimately lead to no change in LT in reduced precipitation plots when grasshoppers are present, as we observed.

Our results show that the presence of a single generalist insect herbivore species can mediate the effect of reduced summer precipitation on plant biomass and functional diversity, but particularly on the CWM of plant functional traits. Future work should incorporate plant functional traits that are physiologically related to plant water use and herbivore resistance such as water‐use efficiency and leaf toughness. The inclusion of trait measurements like water‐use efficiency and leaf toughness when trying to understand the interactive effects of precipitation and insect herbivory would likely improve our ability to mechanistically link the effects of precipitation and insects on communities and ecosystems. However, in our study, the combined effects of reduced precipitation and grasshopper herbivory on the CWM of functional traits demonstrates the importance of common plant species in driving community wide responses to combined abiotic and biotic stressors.

## Author Contributions

**Julia N. Eckberg:** conceptualization (lead), data curation (lead), formal analysis (lead), funding acquisition (lead), investigation (lead), methodology (lead), project administration (lead), visualization (lead), writing – original draft (lead), writing – review and editing (lead). **Mariano A. Rodríguez‐Cabal:** conceptualization (supporting), formal analysis (supporting), funding acquisition (supporting), investigation (supporting), methodology (supporting), project administration (supporting), supervision (supporting), visualization (supporting), writing – review and editing (supporting). **M. Noelia Barrios‐García:** conceptualization (supporting), formal analysis (supporting), funding acquisition (supporting), investigation (supporting), methodology (supporting), project administration (supporting), supervision (supporting), visualization (supporting), writing – review and editing (supporting). **Nathan J. Sanders:** conceptualization (supporting), formal analysis (supporting), funding acquisition (supporting), investigation (supporting), methodology (supporting), project administration (supporting), supervision (lead), visualization (supporting), writing – original draft (supporting), writing – review and editing (supporting).

## Conflicts of Interest

The authors declare no conflicts of interest.

## Supporting information


Appendix S1.

## Data Availability

Data are available from the Environmental Data Initiative (EDI) data portal. DOI: https://doi.org/10.6073/pasta/209b06a1a2933b0f440ea1e9d3130cd0.
