# Supplementary material for: Plant Functional Traits, but Not Community Composition, Are Affected by Summer Precipitation and Herbivory in an Old‐Field Ecosystem
Source: Ecol Evol. 2025 May 7;15(5):e71399. doi: 10.1002/ece3.71399 (PMC12058645; doi:10.1002/ece3.71399)
Supplement: Supplementary file 1 — Appendix S1. [file ECE3-15-e71399-s001.docx]

**Appendix S1**

**Table S1**: Results of t-tests comparing soil volumetric water content (VWC; %) between ambient control and reduced summer precipitation experimental plots every day of experimental rainfall manipulation in 2023. We calculated hourly averages of VWC for each treatment group (control and reduced summer precipitation) prior to running statistical analyses. The t-statistic, degrees of freedom, and p-value are presented for each t-test. P-values were adjusted for multiple comparisons using a Bonferroni correction.

| **Date** | t df p |
| --- | --- |
| 2023-06-28 | -8.74 46 **<0.001** |
| 2023-06-29 | -12.75 46 **<0.001** |
| 2023-06-30 | -10.65 46 **<0.001** |
| 2023-07-01 | -4.81 36 **<0.001** |
| 2023-07-02 | -8.36 44 **<0.001** |
| 2023-07-03 | -6.37 46 **<0.001** |
| 2023-07-04 | -5.3 45 **<0.001** |
| 2023-07-05 | -11.1 43 **<0.001** |
| 2023-07-06 | -2.06 45 1.00 |
| 2023-07-07 | -0.67 45 1.00 |
| 2023-07-08 | 3.31 45 0.09 |
| 2023-07-09 | 4.53 46  **0.002** |
| 2023-07-10 | 7.54 43 **<0.001** |
| 2023-07-11 | 16.3 46 **<0.001** |
| 2023-07-12 | 18.71 46 **<0.001** |
| 2023-07-13 | 12.82 45 **<0.001** |
| 2023-07-14 | 3.06 27 0.23 |
| 2023-07-15 | -1.48 42 1.00 |
| 2023-07-16 | -2.26 40 1.00 |
| 2023-07-17 | 5.09 37 **<0.001** |
| 2023-07-18 | 12.01 46 **<0.001** |
| 2023-07-19 | 19.32 45 **<0.001** |
| 2023-07-20 | 0.57 43 1.00 |
| 2023-07-21 | 6.43 42 **<0.001** |
| 2023-07-22 | 10.93 46 **<0.001** |
| 2023-07-23 | 1.61 42 1.00 |
| 2023-07-24 | 3.19 45 **0.12** |
| 2023-07-25 | 6.63 46 **<0.001** |
| 2023-07-26 | 0.92 44 1.00 |
| 2023-07-27 | 3.75 45 **0.02** |
| 2023-07-28 | 6.54 46 **<0.001** |
| 2023-07-29 | 6.88 46 **<0.001** |
| 2023-07-30 | 6.36 46 **<0.001** |
| 2023-07-31 | 8.34 46 **<0.001** |
| 2023-08-01 | 12.58 46 **<0.001** |
| 2023-08-02 | 12.61 46 **<0.001** |
| 2023-08-03 | 7.52 46 **<0.001** |
| 2023-08-04 | 8.34 46 **<0.001** |
| 2023-08-05 | 6.92 46 **<0.001** |
| 2023-08-06 | 7.62 45 **<0.001** |
| 2023-08-07 | 4.96 45 **<0.001** |
| 2023-08-08 | 1.77 43 1.00 |
| 2023-08-09 | -3.46 46 0.056 |
| 2023-08-10 | -1.08 46 1.00 |
| 2023-08-11 | -1.9 45 1.00 |
| 2023-08-12 | -0.56 45 1.00 |
| 2023-08-13 | 4.77 29 **0.002** |

Abbreviations: t-statistic (t); degrees of freedom (df)

Notes: Bold p-values indicate significance (p < 0.05).


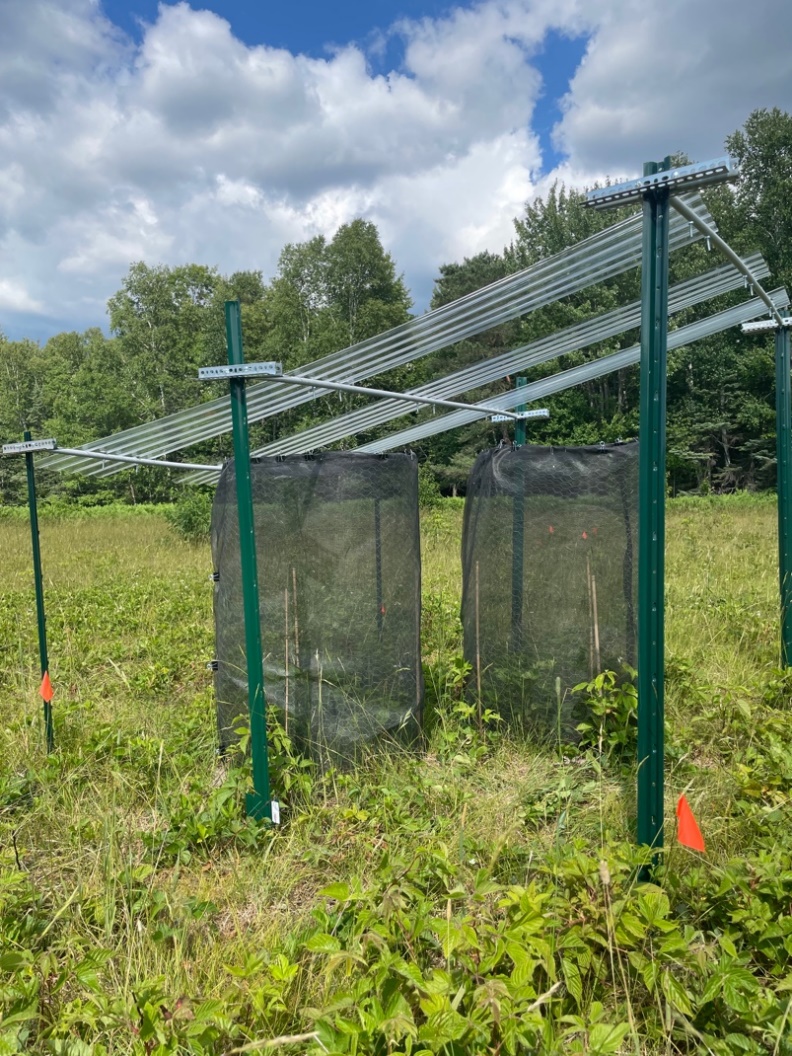


**Figure S1**: Photo of insect enclosures underneath a rainout shelter designed to intercept 50% of incoming precipitation.


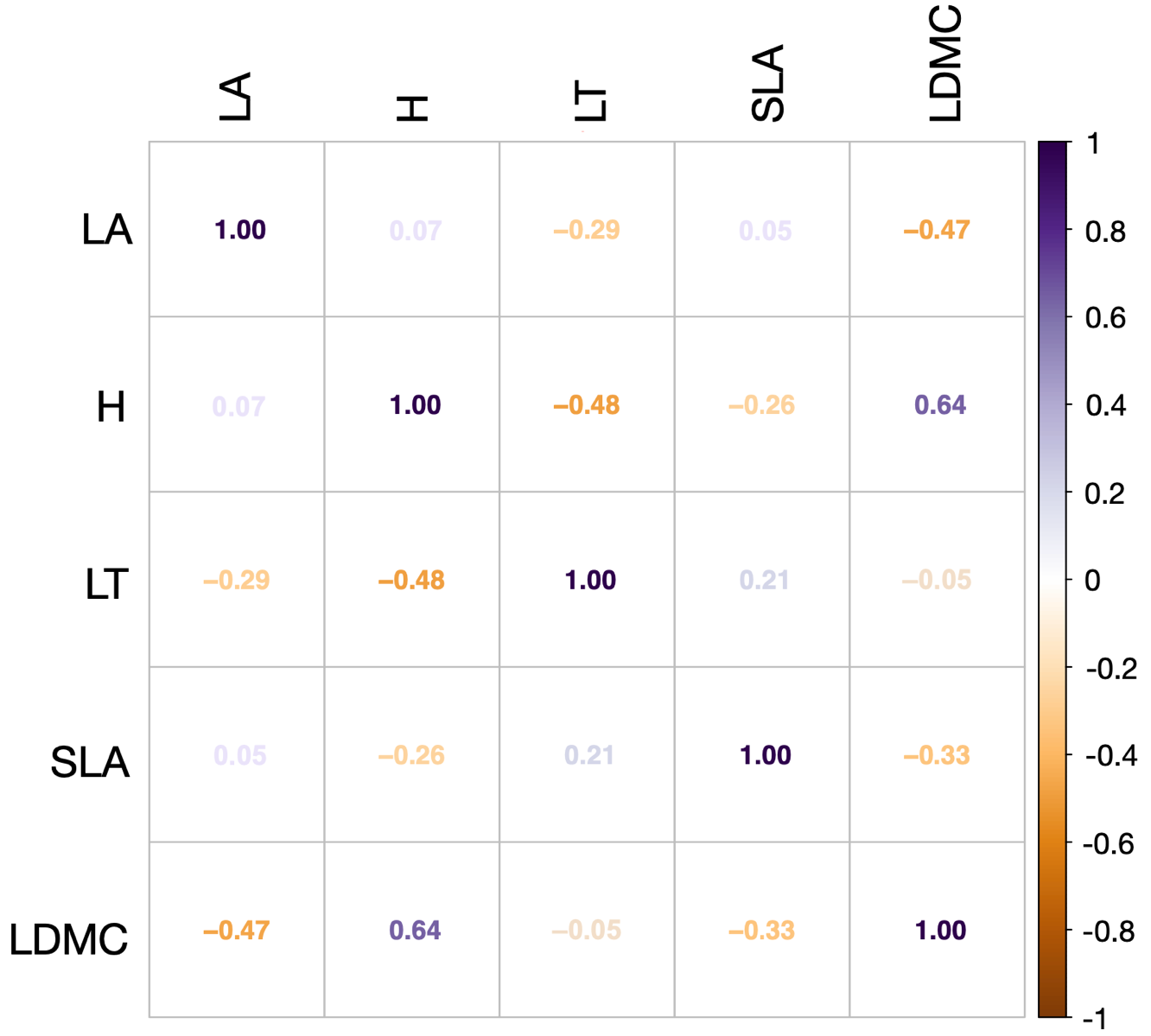


**Figure S2**: Correlation matrix of raw plant functional traits including leaf area (LA; cm^2^), plant height (H; cm), mean leaf thickness (LT; mm), specific leaf area (SLA; cm^2^g^-1^), and leaf dry matter content (LDMC; g g^-1^).

**Figure S3**: Nonmetric multidimensional scaling (NMDS) ordination of plant community composition in 32 insect enclosures. Blue points represent the plant community in enclosures under ambient precipitation plots while the blue ellipse represents the 95% confidence interval around the centroid of ambient precipitation plots. Brown points represent the plant community in enclosures under reduced summer precipitation while the brown ellipse represents the 95% confidence interval around the centroid of reduced summer precipitation plots. Point shape indicates grasshopper presence, with circular points representing enclosures where grasshoppers are absent and triangular points representing enclosures where grasshoppers are present (stress = 0.13; PERMANOVA: F = 0.36, 0.01, p = 0.79; betadisper: F = 0.25, df = 1, p = 0.62).
